# Virus-like particles in poultry disease: an approach to effective and safe vaccination

**DOI:** 10.3389/fvets.2024.1405605

**Published:** 2024-09-09

**Authors:** Abdullahi Abdullahi Raji, Paniz Zarghami Dastjerdi, Abdul Rahman Omar

**Affiliations:** ^1^Laboratory of Vaccine and Biomolecules, Institute of Bioscience, Universiti Putra Malaysia, Serdang, Malaysia; ^2^Department of Veterinary Pathology, Faculty of Veterinary Medicine, Usmanu Danfodiyo University, Sokoto, Nigeria; ^3^Department of Veterinary Pathology and Microbiology, Faculty of Veterinary Medicine, Universiti Putra Malaysia, Serdang, Malaysia

**Keywords:** poultry, vaccine, traditional vaccine, virus-like particles, immunogenicity

## Abstract

The poultry industry, a cornerstone of global food security, faces dynamic challenges exacerbated by viral diseases. This review traces the trajectory of poultry vaccination, evolving from traditional methods to the forefront of innovation Virus-Like Particle (VLP) vaccines. Vaccination has been pivotal in disease control, but traditional vaccines exhibit some limitations. This review examines the emergence of VLPs as a game-changer in poultry vaccination. VLPs, mimicking viruses without replication, offer a safer, targeted alternative with enhanced immunogenicity. The narrative encompasses VLP design principles, production methods, immunogenicity, and efficacy against major poultry viruses. Challenges and prospects are explored, presenting VLP vaccines as a transformative technique in poultry disease control. Understanding their potential empowers industry stakeholders to navigate poultry health management with precision, promising improved welfare, reduced economic losses, and heightened food safety.

## 1 Introduction

The poultry industry plays a significant role in ensuring global food security and nutrition. It provides essential nutrients, energy, and protein to humans, with the ability to convert various agri-food by-products into meat and eggs (1). The industry has been the fastest-growing agricultural sub-sector for the past 50 years, in both developed and developing countries, while rooting deeply into the global food economy (1, 2). The global poultry sector is expected to continue to grow as demand for meat and eggs is driven by growing populations, rising incomes, and urbanization (3). Poultry meat and eggs are vital sources of proteins and other essential nutrients, and smallholder poultry production contributes to poverty reduction and improved food security (4, 5). Additionally, there is potential to use poultry waste to produce value-added products such as fertilizer, biodiesel, animal feed, and biodegradable plastics (6, 7). With its relatively high production, it has the potential to alleviate poverty and contribute to a sustainable and circular economy. However, the industry faces significant challenges due to the outbreak and prevalence of known viral diseases, emerging and re-emerging viral pathogens that can cause substantial economic losses (4, 8).

Major poultry viruses, Marek’s disease virus (MDV), infectious bursal diseases virus (IBDV), infectious bronchitis virus (IBV), avian influenza virus (AIV), infectious laryngotracheitis virus (ILTV), and Newcastle disease virus (NDV), pose major threats to the poultry industry (9–11). These diseases can spread rapidly within flocks, resulting in high morbidity and mortality rates and the ability to emerge and re-emerge with substantial virulence and pathogenic capability (11). These viruses are also highly transmissible and excessively contagious, with extreme transmissibility and the potential for rapid spread across borders, posing significant economic loss, zoonotic potential, and risks to human health (12). To mitigate the outbreak and spread of these viruses at farm-level, national, and international levels (across borders), strategies including vaccination have been employed over the years (13). Vaccination has proven to be an effective strategy for preventing and controlling these diseases, leading to improved flock health and productivity (14).

Poultry vaccination is a fundamental component of modern poultry farming, aimed at preventing and controlling infectious diseases that can significantly impact flock health and productivity. The history of poultry vaccination dates back to 1895 when Louis Pasteur developed the fowl cholera vaccine, and then in the mid-20th century, with the development of vaccines against other avian diseases such as fowl pox, Newcastle disease (ND) and Marek’s disease (14, 15). Over the years, the scope of poultry vaccination has expanded to include a broad spectrum of viral and bacterial pathogens that pose economic threats to the poultry industry. The primary objectives of poultry vaccination are to reduce morbidity and mortality rates, prevent the transmission of pathogens within flocks, and minimize the economic losses associated with diseases (12, 16). Live attenuated vaccines, inactivated vaccines, are the first-generation vaccines and also referred to as classical or traditional vaccines. The second-generation vaccines are the subunit vaccines developed in the 1970s. The third-generation vaccines include the nucleic acid, recombinant vector vaccine, plasmid and mRNA vaccines (15, 17). Each type has its advantages and limitations, influencing their suitability for specific diseases and production systems (14, 15).

Traditional vaccines, such as live attenuated and inactivated vaccines with global significance in terms of acceptance and usage for prophylaxis against important poultry viruses, have come a long way in sustaining the poultry industry (14). Although they have been effective over the years, there are limitations in their long production times, inflexibility in altering antigenic composition, safety concerns, lower efficacy, and higher production costs (18–20). Additionally, the inclusion of an unnecessary antigenic load in conventional vaccines can lead to allergenic and reactogenic responses (21, 22). Inactivated vaccines often require multiple doses and adjuvants to achieve optimal immune responses, while live attenuated vaccines may carry the risk of reversion to virulence (23). New vaccine technology platforms, including DNA, mRNA, recombinant viral vectors (RVVs), and viral-like particle (VLP) vaccines, offer several advantages, such as rapid manufacturing, enhanced efficacy, improved immunogenicity, and cross-protection against multiple strains (15, 24). And in addition, VLP vaccines offer a safer and more targeted immune response, making them a promising alternative to poultry vaccination (25).

The emergence of VLPs has shown promise in veterinary and poultry production and presents a promising alternative to conventional vaccines in poultry production (26). This review aims to provide an analysis of VLP vaccines for poultry disease control. The review will explore the principles of VLP vaccine design, production methods, immunogenicity, and efficacy against major viral diseases affecting poultry. It will also discuss the challenges and future directions associated with VLP vaccine implementation in the poultry industry. By understanding the potential of VLP vaccines and their limitations, researchers and industry professionals can make informed decisions regarding their use in poultry health management. Implementing effective and safe VLP vaccines has the potential to revolutionize poultry disease control, leading to improved animal welfare, reduced economic losses, and enhanced food safety for consumers.

## 2 The evolution of vaccination strategies in poultry

**Figure 1:**
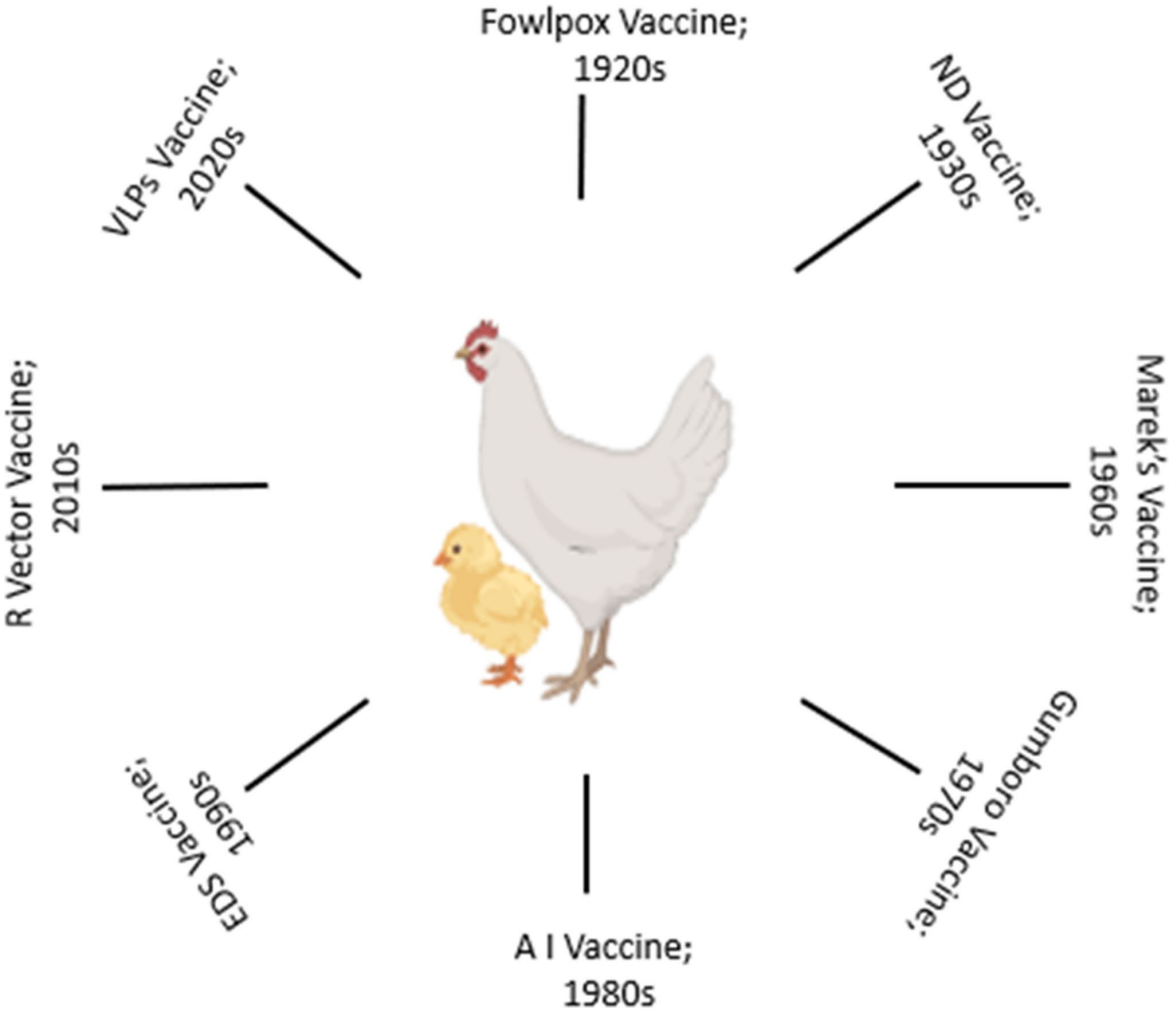
A brief timeline of poultry vaccine evolution.

The evolution of vaccination strategies in poultry is deeply rooted in the historical challenges posed by infectious diseases (Figure 1). In the mid-20th century, the poultry industry faced devastating outbreaks of diseases like ND and Marek’s disease, prompting the development of the first generation of vaccines (27–29). Initial vaccines were often crude and provided limited protection. Over time, advancements in vaccine technologies and scientific understanding have shaped the trajectory of poultry vaccination. Live-attenuated vaccines were among the earliest interventions, leveraging weakened forms of pathogens to induce protective immune responses (14, 27). The classic example is the development of the HVT (herpesvirus of turkeys) vaccine for Marek’s disease (29, 30). While effective, safety concerns regarding the potential reversion to virulence and vaccine-induced disease spurred the exploration of alternative approaches.

The refinement of inactivation techniques led to the development of inactivated vaccines, offering a safer option. The inactivated ND vaccine was a significant milestone in the 1960s, providing an alternative to live vaccines (13, 31). Subunit vaccines, focusing on specific viral components, gained attention, particularly with the advent of molecular biology (32, 33). The late 20th century witnessed the emergence of vector vaccines, where modified viruses or bacteria serve as delivery vehicles for foreign antigens (14, 15). This innovative approach, like other vaccine approaches, allow for the expression of antigens within the host, triggering immune responses. Additionally, DNA vaccines, introduced in the 1990s, marked a shift towards nucleic acid-based vaccines, showing promise in inducing robust immune responses against various poultry pathogens (34). Challenges persisted, including the need for adjuvants to enhance immunogenicity, the emergence of new pathogenic strains, and issues related to vaccine storage and distribution (35, 36). The industry responded with innovations such as oil-emulsion adjuvants, enabling prolonged antigen release, and enhancing vaccine efficacy. Advancements in recombinant DNA technology and protein expression systems allowed for the production of recombinant vaccines. The introduction of recombinant fowl pox vaccines expressing key antigens exemplified the potential for precision in vaccine design (37). The globalization of the poultry industry necessitates strategic vaccination programs. Biosecurity measures, coupled with advances in diagnostics, facilitated a more targeted approach to disease prevention.

## 3 What are VLPs?

**Figure 2:**
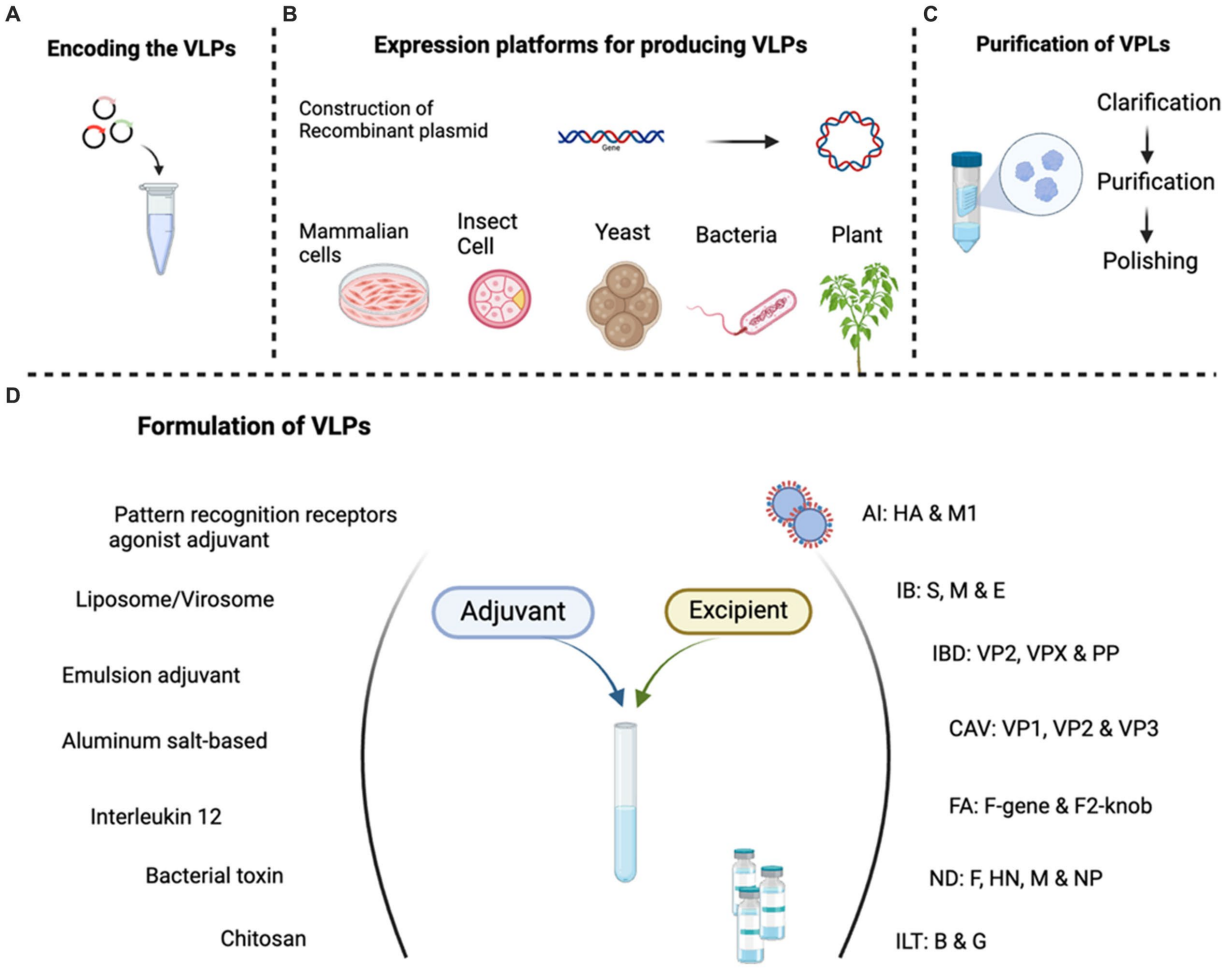
Schematic presentation of VLP vaccine production protocol, created with BioRender.com.

Virus-like particle (VLP) vaccines represent a revolutionary paradigm in vaccinology, offering a sophisticated approach to disease prevention by leveraging the inherent ability of viruses to self-assemble into non-infectious structures. Discovered over 50 years ago, VLPs are self-assembling structures composed of viral structural proteins that mimic the native conformation of the virus without containing the viral genetic material (36). Because the assembled structures lack viral genomes, they are unable to infect or replicate. A variety of eukaryotic and prokaryotic systems are being used to express and purify VLPs (38) (Figure 2). Utilized initially in understanding and resolving the structural architecture of viruses at the atomic level, they have emerged as critical platforms in life sciences (39). Presently, VLPs are utilized in the development of vaccines and as platforms for vaccine production (40). As antiviral vaccines, VLPs have been shown to be highly immunogenic and safe, with the inherent ability to stimulate both innate and adaptive immune responses, mucosal immunity (41–43). In the case of AI for example, VLPs have demonstrated protective efficacy in animal models, inducing neutralizing antibodies and hemagglutination inhibition activities (44). These advantages make VLPs a potential candidate for the development of poultry vaccines, particularly in the context of emerging and re-emerging viral infections. They have garnered significant interest due to their potential advantages over traditional vaccines and even other subunit vaccines (18, 42). VLP vaccines present repetitive antigenic epitopes to the immune system in conformation and move in parallel with protein and viral vector-based vaccines while producing a productive and efficient vaccine (42).

## 4 Structure of VLPs

Structurally, VLPs are created via the spontaneous interaction of viral structural capsid proteins, either singly or in multiples, resulting in the formation of the ultimate structure. They lack a complete virus genome when compared to live viruses. The structural diversity of VLPs renders them highly appealing in terms of both their structural and functional characteristics. Viral capsid proteins can undergo spontaneous polymerization to form VLPs that exhibit geometrical symmetry (36). These VLPs typically take the shape of icosahedral, spherical, or rod-like structures, depending on the specific virus they originated from. VLPs can be categorized into many classes according to their level of structural intricacy. The arrangement of capsid proteins can be classified as one, two, or three layers. Additional monolayer VLPs have the capacity to incorporate several structural proteins. Single-protein VLPs present a simple structure, whereas multi-protein VLPs possess distinctive structural elements, including multiple different capsid layers (36). Some VLPs possess a lipid layer that encompasses viral surface antigens and encloses the capsid structure. This lipid envelope closely resembles the natural infectious virus particle. Another layer of structural classification for VLPs is based on the presence or absence of an envelope, i.e., enveloped or non-enveloped VLPs (45).

Non-enveloped VLPs can be categorized into two groups: single or multi-capsid protein VLPs. Furthermore, VLPs can be classified based on the number of layers they possess, including single-layer, double-layer, or triple-layer VLPs (36). Similar to non-enveloped VLPs, enveloped VLPs are categorized into single-layer, double-layer, and multiple-layer with an internal structure beneath the lipid envelope. During assembly and budding, the cell in which enveloped VLPs are expressed provides their lipid membrane (36). The lipid membrane can accommodate one or more glycoprotein anchors, which are typically the primary antigens recognized by the immune system to generate neutralizing antibodies (36).

## 5 Principles of VLP vaccine design

The development and production of VLP vaccines represent a sophisticated intersection of molecular biology, virology, and biotechnology. This innovative approach leverages the inherent ability of viral structural proteins to self-assemble into particles that mimic the three-dimensional structure of native viruses. The development of vaccines with VLPs against viruses is based on three basic principles; (1) production, (2) purification and (3) formulation (Figure 2). The first step in the development of VLP vaccines is the identification and selection of the viral structural proteins that form the basis of the VLP followed by cloning (36). These proteins are usually the major capsid proteins, such as the envelope glycoproteins or nucleocapsid proteins, depending on the virus of interest (41, 46). These proteins play a crucial role in the self-assembly and integrity of the VLP structure (42, 47).

Once the viral structural proteins have been identified, they are recombinantly expressed using suitable expression systems (36, 48). The selection of an appropriate expression system for VLP production is critical to guaranteeing optimal protein folding and post-translational modifications (PTM) (36). Because of protein PTMs such as glycosylation and phosphorylation, the quaternary structure of viral capsid proteins can alter in different production systems (49). One critical PTM that affects both the stability and immunogenicity of VLPs is glycosylation. Glycan structures on the surface of VLPs assist the immune system recognise VLPs better, leading to a greater antibody response (32, 36). The way that VLPs are absorbed by dendritic cells which are important for triggering immunological responses can be influenced by glycosylation patterns (32, 36). Unique glycosylation patterns are seen in the different expression systems that are employed to produce VLP. Mammalian cell lines, for instance, can produce complicated N-glycosylation akin to that of real viral proteins, which could improve the efficacy of the resultant VLPs. In contrast, lack of glycosylation patterns in bacterial and yeast systems (36, 50), and simpler glycosylation patterns seen in insect systems might influence the stability and immunogenicity of VLPs (23). Plant systems execute specialised glycosylation, which has immunostimulatory effects. Although plant cells lack several mammalian-like PTM changes, the unique glycosylation they give can nonetheless improve the immune response to VLPs produced (23, 51). Differences in glycosylation patterns across production systems can lead to vaccination production consistency and creating difficulties in regulatory approval process. Thus, ensuring that VLPs have the proper glycosylation is central for their safety and effectiveness. And presently, we assume that mammalian and chicken glycosylation patterns are similar. Below is a description of the common expression systems that include insect cells, yeast, plants, mammalian cells, and recently, cell-free expression systems.

### 5.1 Baculovirus/insect cell expression system

The baculovirus/insect cell expression system (B/IECS) is commonly used to produce recombinant proteins in insect cells in high production quantities (14, 52). Baculovirus-based VLP expression is ideal for producing vaccines against viruses with rapidly changing surface antigens during outbreaks (49). It provides many benefits for VLP generation, including the high yield of produced proteins compared to those derived from bacteria or yeast, the presence of complex PTM pathways, and the formation of multi-protein VLPs (32). Conventionally, *Trichoplusia ni* (Tn5) and *frugiperda* (Sf9/Sf21) are used as derivatives in producing recombinant proteins (53). The baculovirus expression vector system (BEVS) has established itself as a production platform for the large-scale production of viral vaccines and gene therapy vectors (54, 55). If not for its production of a simpler N-glycosylation pattern for the expressed glycoproteins, B/IECS would probably serve as the strongest candidate expression system for VLP-based vaccine manufacturing (54).

### 5.2 Plant-derived expression system

Plant-derived VLPs are particularly favorable due to their safety, high expression levels of up to 80% of total soluble protein, immunogenicity, cost-effectiveness, and high-performance expression (33, 36). Over the years, over 55 different plant viruses, including cucumber mosaic virus (CMV), alfalfa mosaic virus (AIMV), potato X virus (PVX), cowpea mosaic virus (CPMV), tobacco mosaic virus (TMV), and papaya mosaic virus (PapMV), have been employed to express antigens on their surfaces. The most commonly used are *Arabidopsis thaliana* and *Nicotiana tabacum* (56). However, only CCMV, CPMV, PVX, and TMV are highly stable at high temperatures and pH levels and are commonly found in plant hosts. Over the years, investigations have demonstrated the protective capacity of plant-expressed VLP vaccines is comparable to or even superior to that of conventional vaccines. In a study, duckweed was used as a medium for the expression of H5 (HA), which was synthesized from an Indonesian H5N1 (A/chicken/Indonesia/7/2003). The recombinant H5 protein vaccine conferred protection after an Intramuscular (IM) administration against the homologous HPAI H5N1 challenge, while the birds remain partially protected against the heterologous viral challenge (57). Utilizing plant viruses to combat a variety of illnesses, such as cancer, infectious disorders, and autoimmune diseases, more than 100 experimental vaccinations have been created in recent years for use in humans and animals, with most at the preclinical stage (36).

### 5.3 *Escherichia coli* expression system

Numerous VLPs are produced by bacteria, which are also one of the most popular expression systems for the synthesis of recombinant proteins. The most typical bacterial host cell for VLP synthesis is *Escherichia coli* (*E. coli*) (36, 58). Numerous benefits of an *E. coli* expression system include low production costs, quick cell growth, high protein expression levels, and ease of scaling up (58). It is frequently recommended to use the *E. coli* expression method to produce tiny proteins with low PTM (59). Of the known chicken *E. coli* VLP vaccines, two are IBD (60) and IB (61), among several others for humans and other animals (36). Of interest, *E. coli* is not a suitable expression system for ND because of its deficiency in the cell membrane and inadequate glycosylation in eukaryotic cells (62). Moreover, a number of chimeric VLP vaccines targeting non-infectious conditions such as hypertension, allergies, diabetes, cancer, and Alzheimer’s disease have been effectively created by linking antigens with bacteriophage Qβ RNA in the *E. coli* expression system (59).

### 5.4 Yeast expression system

Yeast cells are commonly employed for the creation of recombinant proteins and have also been utilized for the production of VLPs (32, 36). The yeast expression platforms, specifically *Pichia pastoris* (*P. pastoris*) and *Saccharomyces cerevisiae*, are highly preferred due to their numerous advantages. These include fast cell growth, high production of expressed proteins, scalability, cost-effectiveness, and the capacity to perform post-translational modification activities (53). Yeast’s capacity for PTMs makes it a significant advancement in VLP synthesis. Several VLPs generated by yeast have already obtained approval from regulatory agencies, including the HPV VLP (63). There is no report yet on yeast cell VLPs in chickens.

### 5.5 Animal cell expression system

Animal cell expression methods are still highly useful and appealing platforms for the production of various structural proteins seen in both non-enveloped and enveloped VLPs (49). Animal cell expression platforms are highly efficient methods for producing recombinant proteins (47, 53). This is because they possess the capability to perform intricate and accurate PTMs, which are crucial for ensuring correct protein folding (58). Avian cell lines have been used with mammalian cell lines for the production of VLPs (64). Nevertheless, the drawbacks of mammalian cell expression methods for producing material for clinical use include limited protein yield, expensive manufacture, lengthy expression time, and the risk of cell lines being infected with mammalian pathogens (53).

## 6 Purification and formulation

Following expression, purification of VLP-based vaccines is a critical aspect of their production, ensuring the removal of contaminants while preserving vaccine integrity (36) (Figure 2). To cut down on the number of stages and expenses involved in the purifying process, a clarification phase is carried out to eliminate aggregates and debris from entire cells that are present in original VLP preparations. The key purification techniques include size exclusion chromatography (SEC), ultracentrifugation (UC), ion exchange chromatography (IEC), and affinity chromatography (AC). Size exclusion chromatography filters particles based on ultracentrifugation leverages centrifugal force; ion-exchange chromatography separates particles based on charge; and affinity chromatography targets specific interactions (54, 65). Challenges in scalability and cost-effectiveness persist, but ongoing research explores innovations such as membrane-based purification and microfluidic systems. Overall, advancements in VLP purification contribute to the potency and safety of these vaccines, marking significant progress in disease prevention strategies (36). Other commonly employed techniques for the removal of components of media or digested DNA or cell debris include diafiltration/ultrafiltration (DF/UF) and tangential flow filtration (TFF) with membranes or hollow fibers (36).

The process of vaccine formulation primarily aims to enhance the stability, effectiveness, and safety of the vaccine during its storage, transportation, and administration. To enhance the effectiveness of VLPs, adjuvants and authorized excipients such as preservatives, buffers, some stabilizing chemical compounds, and stabilizers such as 2-phenoxyethanol, l-histidine, polysorbate 80, and phosphate/sodium borate are commonly included in the majority of vaccine formulations as preservatives, buffering agents, and surfactant stabilizers (66). Several VLPs possess molecular and structural characteristics that can naturally activate the immune system, obviating the necessity for adjuvants. However, the use of adjuvants in VLP vaccine formulations has the potential to enhance the immunogenicity of the vaccine and elicit a targeted immune response (66). Adjuvants such as emulsion adjuvant, pattern recognition receptors (PRRs) agonist adjuvant, aluminum salt-based (Alum) adjuvant, chitosan, bacterial toxin, liposome/virosomes, and interleukin 12 (IL12) are among the several classes of adjuvant that have been tested (66, 67).

## 7 Stability and immunogenicity

Virus-like particles (VLPs) have been engineered to enhance their stability and immunogenicity, making them effective vaccine platforms (68). Compared to subunit vaccines, VLPs are generally considered to be more stable (53). However, changes in environmental conditions, especially during further processing, can lead to significant instability in VLPs due to their lack of the virus’s genetic material. While there are now several VLP vaccines available on the market, some candidates are facing stability concerns (40). In contrast to non-enveloped VLPs, eVLPs are more susceptible to external environmental conditions (53). Various factors, such as temperature fluctuations, shear stress, levels of dissolved oxygen, fluid dynamics, agitation rate, and chemical treatment, can affect the integrity and stability of particles (54). Furthermore, the structural breakdown of eVLPs significantly reduces their immunogenicity and disrupts cellular proliferation and the synthesis of metabolic proteins, thus impacting VLP production. This has been identified as one of the primary challenges in using eVLPs as a substitute for live viruses in vaccine production. However, many modifications, such as the insertion of stabilizing mutations, have been made to enhance their thermal stability (53). These stable and versatile nanoparticles have been shown to induce potent humoral and cellular immune responses, making them a safe and effective alternative to inactivated infectious viruses (45). Previous studies have shown that VLPs have the potential to provide greater immunogenicity and antigenicity compared to subunit vaccinations (32). The efficacy of these particles has the capacity to greatly stimulate cellular and humoral immunity (40). Following the VLPs, the dendritic cells (DCs) exhibit the expression of different maturation markers such as CD40, CD80, and CD86. These markers play a crucial role and are expressed on the surface of DCs upon activation and play a role in the subsequent activation of T cells. Initially, DCs are stimulated by attaching VLPs to a certain pattern seen on the surface of DCs known as PRRs, specifically TLR (69). Then, VLPs are internalized inside the cytosol of DCs and are then displayed to cytotoxic T cells and helper T cells by MHC class I and class II molecules, respectively (70). VLPs have the ability to not only activate B cells to initiate an antibody response, but they can also induce the proliferation of CD4^+^ T helper (TH) and CD8^+^cytotoxic T cells (36).

## 8 Efficacy and experimental studies

Although all documented poultry VLP vaccines are still been studied at different experimental levels, the evaluation of efficacy through well-designed trials is a crucial step in establishing the practical utility of VLP vaccines in the poultry industry. Numerous studies have delved into experimental assessment of the effectiveness of VLP vaccines in protecting poultry populations against a spectrum of avian diseases. Field trials play a pivotal role in evaluating the real-world efficacy of VLP vaccines in poultry. These trials involve administering the VLP vaccine to large populations of birds under natural conditions, allowing researchers to observe its performance in diverse environments. Experimental studies are been conducted and still ongoing for various avian diseases, such as AI, ND, IBD, and IB, showcasing the practical effectiveness of VLP vaccines in controlling outbreaks and minimizing economic losses (33, 44, 71, 72). Controlled challenge studies are fundamental to understanding the protective capacity of VLP vaccines against specific avian pathogens (72, 73). In these experiments, vaccinated birds are intentionally exposed to the target pathogen, and the degree of protection is meticulously assessed. Such studies provide valuable insights into the ability of VLP vaccines to induce immunity and reduce the severity of disease in poultry (33).

Furthermore, comparative efficacy assessments involve benchmarking VLP vaccines against existing conventional vaccines or alternative strategies. These studies aim to demonstrate the superiority or comparable effectiveness of VLP vaccines in eliciting protective immune responses in poultry. Comparative trials contribute to establishing VLP vaccines as viable alternatives with distinct advantages, such as broad-spectrum protection and improved safety profiles (36).

## 9 Application of VLP vaccines in disease prevention in poultry

The use of VLP vaccines in poultry disease prevention has been explored in various studies and represents a groundbreaking approach with significant potential to enhance the health and productivity of avian populations. Extensive research and trials have elucidated the effectiveness of VLP vaccines against some important poultry diseases, including IBD, IB, AI, and ND, providing a novel avenue for proactive disease management (33, 61) (Table 1). These vaccines offer promising benefits such as nonreplicating and able to elicit mucosal immunity which is lacking in killed conventional vaccine, reduced virus shedding and the ability to differentiating infected from vaccinated animals (DIVA) birds, and versatile routes of administration (33, 36, 74). Furthermore, the targeted delivery of antigens to antigen-presenting cells has been proposed as a strategy to enhance the protective efficacy of poultry vaccines, including VLP vaccines (75).

**Table 1:** Chicken VLPs based vaccine candidates in preliminary stages of experimental studies.

S/No	Virus/disease	Composition (protein)	Stage of development	Platform/expression system	References
1	Infectious bronchitis	S, M, E	Research stage/preliminary stages of animal testing	Plant BVIC	(61, 96, 97)
2	Infectious laryngotracheitis	B, G	Research stage/preliminary stages of animal testing	LMH	(98, 99)
3	Avian influenza	HA, M1	Research stage/preliminary stages of animal testing	BVIC Plant	(33, 71, 100–105)
4	Chicken anemia virus	VP1, VP2, VP3	Research stage/preliminary stages of animal testing	BVIS	(106, 107)
5	Infectious bursal disease	VP2, VPX, PP	Research stage/preliminary stages of animal testing	BVIC *E. coli* Plant Yeast	(35, 60, 108–111)
6	Newcastle disease	F, HN, M, NP	Research stage/preliminary stages of animal testing	Plant BVIC	(26, 33, 62, 112–115)
7	Fowl adenovirus	Fiber gene, F2-knob	Research stage/preliminary stages of animal testing	*E. coli* BVIC	(116–119)

### 9.1 Avian influenza

Avian influenza poses a constant threat to poultry worldwide, with the potential for severe economic repercussions. In the case of AI, VLPs present key viral antigens, such as hemagglutinin (HA) and neuraminidase (NA) in a conformation that closely resembles the native virus (76). This structural similarity enhances antigenic presentation, leading to the induction of potent and specific immune responses in vaccinated poultry, conferring protection against diverse strains of AI (71, 77). Breakthroughs in VLP vaccines against AI H5 in chickens involve the development of a bivalent H5 and H7 VLP vaccine. This vaccine, created using a baculovirus expression system, demonstrated immunogenicity and protection against H5N1 and H7N9 viruses in chickens. Compared to the commercially available whole-virus inactivated vaccine, the bivalent VLP vaccine expresses the capability to DIVA due to the presence of non-structural protein (NP) antigens (78). Other expression systems have been used to make the VLPs against AI too; for instance, plant-based VLPs vaccine was produced against H5 and H9 subtypes of the AI virus using *Nicotiana benthamiana* as an expression system (79).

### 9.2 Infectious bronchitis

Infectious bronchitis is a highly contagious respiratory disease in chickens, causing significant economic losses in the poultry industry (11, 80). The disease also has the potential to destroy the oviducts and kidneys (80, 81). IBV exhibits a high degree of genetic variability, leading to the emergence of multiple strains, with little or no cross-protection against distinct variant, making its control extremely difficult. VLP vaccines offer the advantage of inducing cross-protection, meaning they can confer immunity against various IBV strains. This cross-protection is essential for controlling the disease effectively, especially in regions where multiple IBV variants are prevalent. VLP vaccines designed for IBV focus on presenting key viral envelope proteins, such as the spike (S) protein. The S protein is a major target for inducing immune responses as it plays a critical role in viral entry and is a major inducer of protective immunity. These vaccines stimulate strong humoral immune responses, leading to the production of neutralizing antibodies. Similarly, VLP vaccine presenting S, E, and M proteins of IB expressed in recombinant baculovirus has been shown to be a promising vaccine candidate for IB, as it can stimulate strong immune responses and provide effective protection (61). Field trials and experimental studies have demonstrated the practical efficacy of VLP vaccines in reducing clinical signs associated with IBV infection. Vaccinated birds exhibit milder respiratory symptoms, decreased mortality rates, and reduced viral shedding compared to their non-vaccinated counterparts. This reduction in viral shedding contributes to limiting the spread of the virus within poultry populations (61). VLP vaccines for IBV can be integrated into combination vaccines, protecting against multiple poultry pathogens in a single formulation. This approach streamlines vaccination programs, reduces stress on birds, and enhances overall disease prevention strategies in poultry farming (61).

### 9.3 Newcastle disease

Newcastle disease, caused by avian orthoavulavirus 1 (OAV-1), remains a global concern for poultry health (82, 83). VLP vaccines expressing M-protein as the skeleton, while the F and HN proteins (protective antigens) were displayed on the surface. The F-protein plays a pivotal role in virus entry and membrane fusion. VLP vaccines expressing the F-protein induce the production of neutralizing antibodies, preventing the virus from entering host cells have demonstrated efficacy in reducing clinical signs and mortality associated with NDV infection (62). Experimental studies and field trials have shown that vaccinated birds exhibit a longer protection period, milder clinical symptoms, less tissue load, lower mortality rates, and decreased viral shedding compared to their non-vaccinated counterparts (62). Trials have also demonstrated the induction of both humoral and cell-mediated immune responses, contributing to the establishment of a robust defense against the Newcastle disease virus strain in chickens (62). This suggests the potential of NDV VLPs as an alternative to current live genotype-unmatched vaccines for controlling and eliminating NDV in poultry flocks. The study by Park et al. (84) also highlights the significance of DIVA using the hemagglutination inhibition (HI) test, which is crucial for effective vaccination programs. The results indicated that a single immunization with 10 or 50 μg of NDV VLP vaccine could fully protect chickens after a lethal NDV challenge and effectively reduce challenge virus shedding. The successful DIVA test performed with the HI test supports the potential application of VLP vaccine as part of an NDV control strategy,

In a study, the ND F and/or HN proteins of a genotype VII.2 strain were put into a VLP that was grown in *Nicotiana benthamiana*. After the *in vivo* trial, the results showed high seroconversion in the SPF chickens after a single dose of the VLP vaccine. Next, in an *in vitro* experiment, the VLP was able to successfully inhibit the viral replication of two antigenically closely related ND virus isolates. This presents a great opportunity for the poultry industry to have an antigen-matched vaccine that is highly immunogenic and cost-effective.

### 9.4 Infectious bursal disease

Infectious Bursal Disease (IBD), caused by the infectious bursal disease virus (IBDV), is a highly contagious and immunosuppressive disease affecting young chickens (60, 85). In 2013, the first candidate multivalent VLP vaccine was experimentally developed against both a variant (VarIBDV) strain (USA08MD34p) and a classic IBDV strain (Mo195). The vaccine was created by co-expressing two separate structural proteins (pVP2 and VP3) and co-infecting insect cells. Two vectors, pVP2 and VP3, were cloned from the variant strain, while pVP2 was cloned from the classical strain. This multivalent VLP vaccine was capable of stimulating the immune system and possessed the antigenic integrity of both the variant and classic viruses. Consequently, it elicited a robust humoral immune response and protected chickens against the virus (86). Subsequently, in 2015, Lee et al. demonstrated a VLP vaccine candidate against the LC10 strain, which is a very virulent IBDV (vvIBDV) isolated from Korean broilers. A single recombinant baculovirus insect cell was used to co-express the precursor polyprotein (PP) and VP4. The promising results of the VLP vaccines lead to another VLP vaccine candidate. In 2021, Wang et al. developed a VLP candidate vaccine against a novel (nVarIBDV), a significant threat to the poultry industry (60, 73). The VP2 protein of nVarIBDV was successfully expressed and purified, leading to the self-assembly of 25-nm VLPs.

## 10 Challenges and considerations

The integration of VLP vaccines into poultry health management, while promising, brings forth a set of challenges and considerations that require careful examination (14). Poultry VLP vaccines gaining significant attention as a potential solution for combating viral diseases in poultry. One of the main challenges of poultry VLP vaccines is the complexity of vaccine development (87). Creating effective VLP vaccines requires a thorough understanding of the target virus as well as the ability to engineer and produce the VLPs. Additionally, ensuring that the VLP is stable and can induce a strong immune response in poultry presents another hurdle. Stability in this context refers to the ability of the VLPs to maintain their structure and function during storage and transportation. Achieving stability is crucial for ensuring that the vaccine remains potent and effective when administered to poultry (14, 36).

Furthermore, stable VLP contributes to the overall safety and efficacy of the vaccine, making it a key consideration in the development process. Moreover, the selection of appropriate adjuvants for poultry VLP vaccines is crucial (88). Adjuvants play a crucial role in enhancing the immune response to the vaccine, but selecting the right adjuvant for poultry VLP vaccines requires thorough research and testing to ensure both effectiveness and safety (36, 67). Additionally, the formulation of the adjuvant with the VLPs must be carefully optimized to achieve the desired immune response without compromising the stability and integrity of the vaccine. The immune response in poultry may differ from that of other animals, so finding adjuvants that are effective and safe for use in poultry can be challenging (89). Another challenge in the development of poultry VLP vaccines is the need to accurately target specific serotypes of viruses. This is especially important for poultry diseases such as AI, IB, IBDV, and ND that have multiple serotypes, as the vaccine needs to be able to protect against all of them (14). Similar to this, factors like the strain and variability of the target virus can affect how effective poultry VLP vaccines are (90).

The transition from laboratory-scale to large-scale production of VLP vaccines introduces inherent challenges that merit attention. Optimizing production systems and ensuring consistent VLP quality are critical considerations, with scalability being a key factor in large-scale manufacturing (23). Additionally, the logistics of distribution and storage present challenges that need meticulous planning to maintain VLP vaccine integrity (23, 36). Overcoming these challenges is essential to achieving cost-effective large-scale production and establishing robust distribution networks, ultimately impacting the accessibility and affordability of VLP vaccines for poultry producers (14). In addressing these challenges, collaborative efforts among researchers, vaccine manufacturers, and regulatory bodies become paramount (18). By collectively tackling these challenges, the poultry industry can unlock the full potential of VLP vaccines, revolutionizing disease prevention strategies and enhancing the overall health and productivity of poultry populations.

## 11 Future perspectives

As an emerging and promising alternative to traditional vaccines in the field of poultry vaccination, one potential future perspective on poultry VLP vaccines is the development of non-injectable vaccine delivery methods. This could involve the use of oral, intranasal, or topical application of the vaccine, which would offer a more convenient and less stressful method of administration for both poultry producers and the birds themselves. Additionally, the possibility of not using adjuvants in VLP vaccines could be explored, potentially reducing the risk of adverse reactions, and simplifying the vaccination process. These advancements could pave the way for safer and more accessible poultry vaccination programs, ultimately benefiting the poultry industry. Another future perspective for poultry VLP vaccines is their potential use in controlling and eradicating avian diseases through the implementation of DIVA strategies (33, 91). Research and development efforts are also focused on enhancing the stability and shelf-life of poultry VLP vaccines, ensuring their effectiveness under various storage conditions. Moreover, there is a growing interest in the use of VLP vaccines for combating emerging and re-emerging infectious diseases in poultry, providing a proactive approach to disease prevention (42).

Another notable prospect is their potential role as delivery vehicles for therapeutic agents or platforms for targeted drug delivery in poultry. The immunomodulatory properties of VLPs suggest broader applications, possibly contributing to overall immune health and well-being in poultry beyond protection from specific diseases (92). Aligning with the principles of precision medicine, VLP vaccines could play a pivotal role in personalized approaches to poultry health. The ability to customize VLPs based on individual genetic or environmental factors influencing disease susceptibility holds promise for optimizing vaccine efficacy (93, 94). Integrating genomic information into vaccine strategies could lead to tailored solutions for individual poultry populations, contributing to a more precise and effective disease prevention approach. Looking ahead, the trajectory of VLP vaccine production in poultry health is poised for continual advancements and innovations. The integration of cutting-edge technologies, such as CRISPR/Cas9 for precise genetic engineering of VLPs, holds promise for enhancing vaccine immunogenicity (120). Additionally, exploring novel adjuvants and delivery systems may further contribute to improved vaccine performance. Collaborative efforts among researchers, biotechnologists, and poultry industry stakeholders will be pivotal in driving innovations and ensuring the continued evolution of VLP vaccine technology (36).

Overall, the future of poultry VLP vaccines is brimming with possibilities, from innovative delivery methods to tailored and multi-valent vaccines, all contributing to the advancement of poultry health and welfare (17). Considering the accumulated knowledge of pathogenesis, immune response, and prerequisites for protective immunity against poultry diseases, future perspectives on poultry VLP vaccines are focused on addressing the existing challenges and limitations of conventional vaccines, such as safety concerns and reduced immune response (17, 36). With ongoing research and development efforts, the use of VLP vaccines in poultry is expected to expand significantly in the coming years (25). The potential of poultry VLP vaccines goes beyond disease prevention in poultry populations; they also have implications for human health (17). One exciting area of potential for poultry VLP vaccines is their use in zoonotic disease prevention. Zoonotic diseases are pathogens that can be transmitted from animals to humans, and given the close interaction between poultry and humans in various agricultural and domestic settings, the development of VLP vaccines for poultry could have significant implications for public health (17, 23).

## 12 Conclusion

In the realm of poultry health, the advent of VLP vaccines marks a revolutionary stride towards more effective and versatile disease prevention strategies. Despite these challenges, the future of VLP vaccines in poultry disease control appears promising. Continued research and innovation are necessary to optimize production methods, improve vaccine formulation, enhance stability, and address regulatory considerations. Additionally, exploring novel approaches, such as the use of alternative expression systems or combination vaccines, will contribute to the broader application of VLP vaccines in the poultry industry. Overall, VLP vaccines hold tremendous potential for protecting poultry from viral diseases, minimizing economic losses, and ensuring food safety. With further advancements and collaborations between researchers, industry, and regulatory authorities, VLP vaccines can contribute significantly to the sustainable and effective management of viral diseases in poultry populations.
